# Mechanical stability of talin rod controls cell migration and substrate sensing

**DOI:** 10.1038/s41598-017-03335-2

**Published:** 2017-06-15

**Authors:** Rolle Rahikainen, Magdaléna von Essen, Markus Schaefer, Lei Qi, Latifeh Azizi, Conor Kelly, Teemu O. Ihalainen, Bernhard Wehrle-Haller, Martin Bastmeyer, Cai Huang, Vesa P. Hytönen

**Affiliations:** 1 Faculty of Medicine and Life Sciences and BioMediTech, University of Tampere, Finland and Fimlab Laboratories, Tampere, Finland; 2 Zoological Institute, Cell- and Neurobiology, Karlsruhe Institute of Technology (KIT), Institute of Functional Interfaces (IFG), Karlsruhe, Germany; 3 Markey Cancer Center and Department of Molecular and Biomedical Pharmacology, University of Kentucky, Lexington, KY USA; 4 Department of Cell Physiology and Metabolism, University of Geneva, Geneva, Switzerland

## Abstract

Cells adhere to the surrounding tissue and probe its mechanical properties by forming cell-matrix adhesions. Talin is a critical adhesion protein and participates in the transmission of mechanical signals between extracellular matrix and cell cytoskeleton. Force induced unfolding of talin rod subdomains has been proposed to act as a cellular mechanosensor, but so far evidence linking their mechanical stability and cellular response has been lacking. Here, by utilizing computationally designed mutations, we demonstrate that stepwise destabilization of the talin rod R3 subdomain decreases cellular traction force generation, which affects talin and vinculin dynamics in cell-matrix adhesions and results in the formation of talin-rich but unstable adhesions. We observed a connection between talin stability and the rate of cell migration and also found that talin destabilization affects the usage of different integrin subtypes and sensing of extracellular matrix proteins. Experiments with truncated forms of talin confirm the mechanosensory role of the talin R3 subdomain and exclude the possibility that the observed effects are caused by the release of talin head-rod autoinhibition. In conclusion, this study provides evidence into how the controlled talin rod domain unfolding acts as a key regulator of adhesion structure and function and consequently controls central cellular processes such as cell migration and substrate sensing.

## Introduction

Cell-matrix adhesions are large and dynamic membrane spanning protein complexes that physically anchor animal cells to their environment. These complexes connect integrin adhesion receptors to actin fibers providing a mechanical link between the cytoskeleton and the extracellular matrix. In addition to mechanical force, cell-matrix adhesions transmit biochemical signals across the plasma membrane and they have an important role in the regulation of cell anchorage, spreading and migration. The central role of cell-matrix adhesions in force transmission also makes them hotspots for cellular mechanotransduction. Mechanotransduction describes the cellular processes that translate mechanical tension or forces into a chemical or electrical signal. These processes allow cells to probe the mechanical properties of the surrounding tissue and to react to forces exerted on them^1^. Mechanotransduction regulates many processes on the levels of individual cells and complete tissues and it is involved in the development and progression of various diseases^2^. Despite the intense research focusing on the mechanotransduction of cell-matrix adhesions, the primary mechanosensory proteins in these adhesions remain largely unknown.

Talin is a 270 kDa adhesion protein containing a globular N-terminal head domain and a C-terminal rod domain composed of a series of alpha-helical bundles. The head domain (47 kDa) contains binding sites for multiple adhesion proteins and its binding to the β-integrin tail is one of the first steps in the formation of nascent cell-matrix adhesions. The head domain is linked to the rod domain by an unstructured linker region (9 kDa) which, when fully extended, increases the length of the protein by 20 nm and contains a protease cleavage site involved in adhesion turnover^3, 4^. Talin rod domain (~210 kDa) consists solely of alpha-helices, assembled into 13 subdomains. Each subdomain contains 4 to 5 amphipathic helixes folded into a compact helix bundle with a hydrophobic core. Talin rod subdomains have binding sites for other adhesion proteins, including vinculin, Rap1-GTP-interacting adapter molecule (RIAM), Deleted in liver cancer 1 (DLC1), β-integrins and actin, as reviewed by Calderwood *et al*.^5^. The most numerous binding sites are for vinculin, with a total of 11 binding sites located in 9 of the 13 rod subdomains. Many of these vinculin binding sites (VBS) are cryptic, which means that vinculin can only bind to them after the subdomain has partially unfolded. This unfolding event is thought to be initiated by mechanical force transmitted across the rod domain^6, 7^. Different talin rod subdomains have been shown to unfold at different forces, ranging from 5–10 pN to 25–40 pN in *in vitro* experiments^8–10^. The gradual force-induced exposure of the talin VBSs creates a system where higher force causes more rod subdomains to unfold, exposing more VBSs. Vinculin accumulation is known to not only mechanically strengthen the adhesion, but also to initiate downstream signaling cascades. In addition, such multi-step unfolding of the talin rod domain has been suggested to create a force buffer that can smooth out sudden fluctuations in the cellular traction forces^9^.

Talin is among the first proteins involved in integrin-mediated adhesion formation^11^. Therefore, mechanotransduction by the force-induced unfolding of talin rod subdomains may have an important role in promoting either maturation or disassembly of nascent adhesions^11, 12^. The R3 subdomain of talin has been found to be the first subdomain to open under mechanical load, unfolding in *in vitro* experiments already at a 5 pN pulling force^8–10^. This low mechanical stability of the R3 subdomain makes it especially suitable for acting as a mechanosensor during adhesion maturation, where low magnitude forces are transmitted through the talin rod domain. If the force-induced unfolding of the talin R3 subdomain is a key step in adhesion maturation, mechanically stabilizing or destabilizing mutations should affect cellular mechanosensing and mechanosignaling. In a previous study, mechanically stabilized talin R3 subdomain was found to affect fibroblast substrate rigidity sensing and YAP signaling, highlighting the importance of talin R3 subdomain in mechanosensing^13, 14^. However, this experiment does not give any indication about whether also destabilization of talin R3 subdomain would result in altered mechanosignaling and changes in cell phenotype.

In this study, we present a series of talin point mutations that destabilize the talin rod R3 subdomain. Steered molecular dynamics simulations were used to confirm that these mutations decreased the mechanical stability of the R3 subdomain. Expression of these talin mutants in fibroblast cells enabled us to study the importance of this rod subdomain for talin recruitment into cell-matrix adhesions. Importantly, we show that even a single destabilizing mutation affected adhesion protein composition and adhesion dynamics. On a cellular level, talin destabilization decreased both the cell migration rate and traction force generation and affected integrin subtype usage and ECM sensing. This study demonstrates that a controlled decrease in the stability of talin rod domain affects several cellular functions and sheds light into the importance of talin stability as a key regulator of adhesion mechanosignaling.

## Results

### Stepwise mutagenesis destabilizes the talin R3 subdomain in steered molecular dynamics simulations and affects subdomain folding *in vitro*

**Figure 1:**
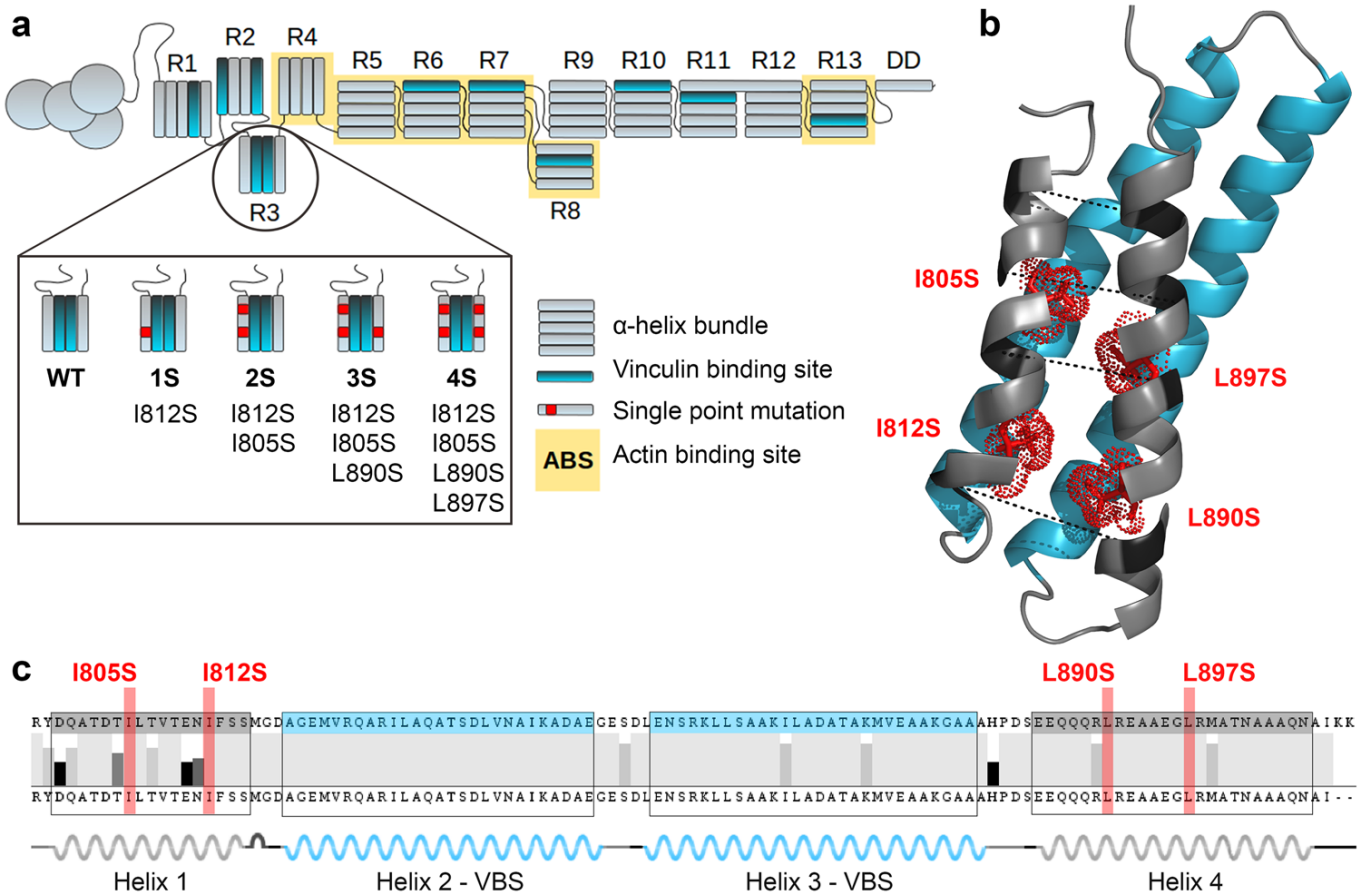
Talin-1 R3 domain destabilization by mutagenesis. (**a**) Schematic illustration of wild-type talin-1 and the locations of the mechanically destabilizing point mutations in the R3 subdomain in mutants 1S, 2S, 3S and 4S. (**b**) A cartoon model (PDB ID: 2L7A^14^) of mouse talin-1 R3 domain. The four mutated residues are shown as stick models colored in red and indicated with red labels. The pulling vectors in the SMD simulations are marked with a dashed black line and respective amino acids are indicated with dark coloring. The two vinculin binding helices in the R3 subdomain are colored in blue. (**c**) Talin-1 R3 domain helix boundaries and sequence conservation across 13 animal species (Human gi6739602, Mouse gi227116327, Rat gi189181726, Hamster gi344251776, Naked mole rat gi351707040, Threeshrew gi444729903, Bat gi432110771, Flying fox gi431902812, Cattle gi296484714, Turtle gi465952424, Quail gi667665823, Chicken gi45383127, Zebrafish gi57222259). The positions of the two VBS helices and four destabilizing point mutations are colored in blue and red, respectively.

Talin-1 subdomain R3 is a four helix bundle located within the compact N-terminal end of the talin rod domain. It contains 2 vinculin binding sites in the helices 2 and 3 (Fig. 1b,c). Due to the presence of hydrophilic threonine residues in the hydrophobic core, the mechanical stability of the R3 subdomain helix bundle is relatively low^8^. With our mutagenesis design, we aimed to further destabilize the R3 subdomain by mutating conserved isoleucine and leucine residues within the hydrophobic core of the helix bundle to a small polar amino acid, serine. This addition of hydrophilic residues into the core of the helix bundle makes its tertiary structure thermodynamically less favorable, and thus facilitates its unfolding when the helix bundle is subjected to mechanical stretching. The multiple sequence alignment presented in Fig. 1c shows that the residues selected for mutagenesis are highly conserved over animal species. The side chains of the selected hydrophobic residues are oriented towards the core of the helix bundle (Fig. 1b). Furthermore, the residues targeted for mutagenesis are in a belt or ladder-like assembly through the hydrophobic core at sufficient distance from each other as well as from other polar residues in the core, so that stabilization of the mutated helix bundle through the organization of polar planes is unlikely. After the identification of the potential target residues for mutagenesis, a panel of mutants containing one to four single point mutations I805S, I812S, L890S and L897S in each construct was prepared (Fig. 1a).

**Figure 2:**
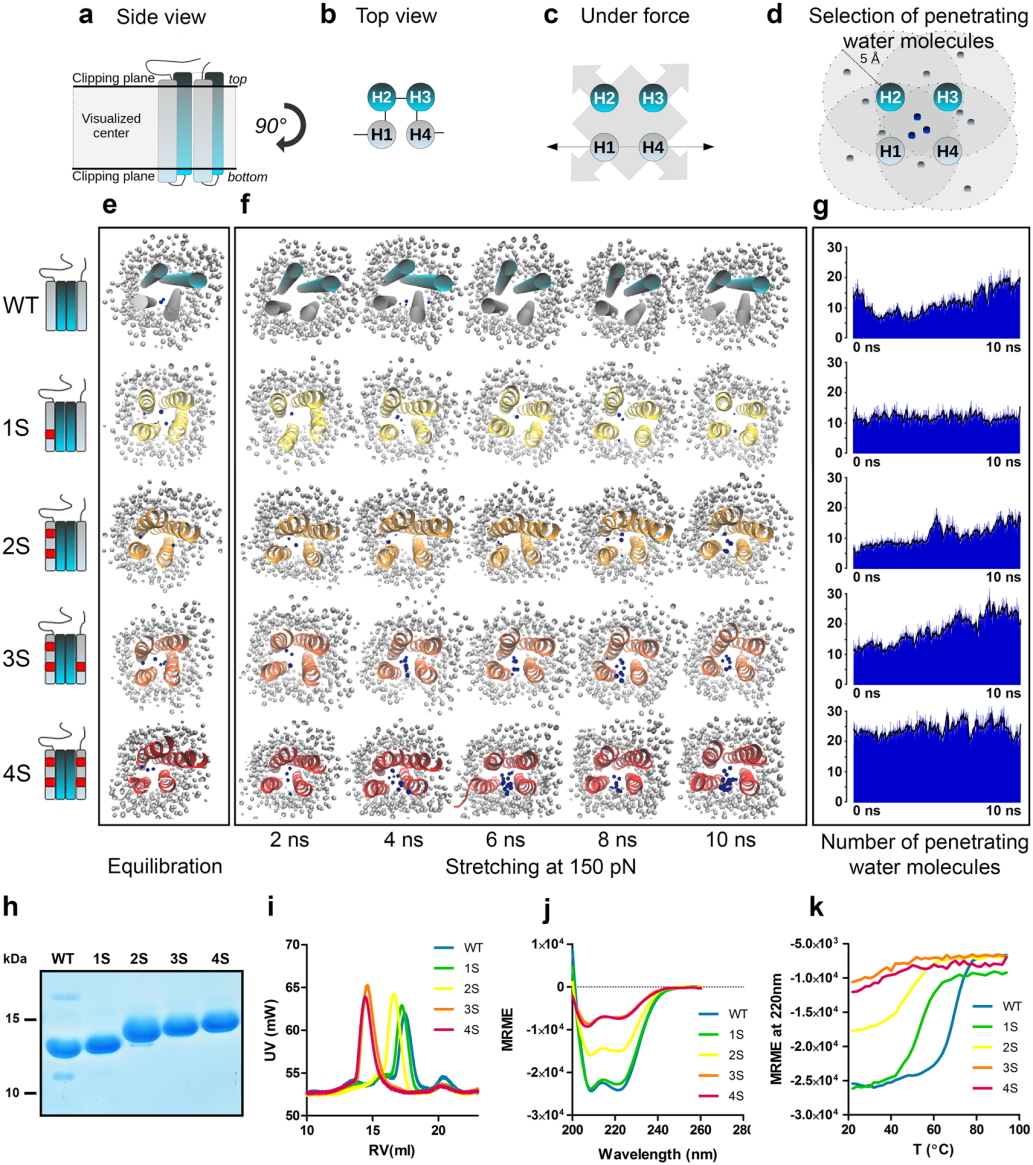
Simulated mechanical stretching and biochemical characteristics of destabilized mouse talin-1 fragments. Talin R3 subdomain stability during simulated mechanical stretching was analyzed by observing water penetration into the hydrophobic core of the subdomain. VBS in cyan color, point mutations in red, non-penetrating water in gray, penetrating water in blue. (**a–d**) Schematics of talin R3 subdomain helix positions and the criteria for selecting water molecules penetrated into the bundle core. The clippings planes used in visualizations in (**e**,**f**) are shown in (**a**). In (**d**), water molecules within 5 Å of each helix are marked in blue. (**e**) Snapshot images of the destabilized talin-1 R3 subdomains after a 20 ns equilibration without pulling force. The planes above and below the bundle ends are hidden to allow unobstructed view into the bundle core (schematics in (**a**)). Penetrating water molecules are colored in blue while water molecules outside the bundle are colored in gray. (**f**) Representative serial snapshot images of talin-1 R3 subdomain opening during a 10 ns simulation with a constant force of 150 pN. (**g**) The number of penetrating water molecules during a 10 ns pulling simulation as an average count of 5 experiments at each time point. Black lines represent sliding averages of 10 frames. Water molecules at the ends of the bundle are included in this total count, although they are hidden from the snapshot images in (**e**,**f**). (**h**) PageBlue staining for destabilized talin R3 subdomain fragments (amino acids 795 – 911) expressed in *E*. *coli*, affinity purified and separated by SDS-PAGE in the presence of 8 M urea. Apparent differences in fragment sizes result from differential SDS binding to purified talin forms. (**i**) Size exclusion chromatography analysis for the R3 subdomain fragments. RV = Retention volume (**j**) Circular dichroism spectroscopy scanning for the talin R3 fragments at wavelengths 200 nm–260 nm. MRME = Mean residue molar ellipticity. (**k**) Circular dichroism spectroscopy melting curves at 220 nm wavelength. Temperature was increased in 2 °C steps at a rate of 1 °C/min. Determined melting points for WT and 1S – 4S mutants were 66.9 °C, 51.6 °C, 48.0 °C, 40.7 °C and 40.9 °C, respectively.

The destabilized point mutations were investigated by using constant force steered molecular dynamics (SMD) simulations. The bundle behavior was assessed by analyzing the number of water molecules penetrating into the hydrophobic core of the R3 subdomain (Fig. 2). SMD simulations showed decreasing mechanical stability under mechanical load with the increasing number of single point mutations in the talin R3 bundle, as can be observed on the penetrating water count in Fig. 2g. The increase in the number of the penetrating water molecules reflects an increase in the total volume of the bundle due to the acting force and correlates with the number of destabilizing mutations. With the increased volume and area, also the number of water molecules that comply with the penetrated water selection criteria increases. The effect of the mutations on the R3 subdomains ability to withstand mechanical forces was also analyzed by observing the distance between the helix 1 (H1) and the helix 4 (H4) (Supplementary Figure S1). The H1-H4 displacement over time was followed on four vectors, drawn between the α-carbons of residues Gln800 – Ala904, Thr804 – Ala900, Val808 – Gly896, and Ser815 – Gln888. During 10 ns simulations at 150 pN, WT and 1S mutant subdomains underwent only negligible changes in the range of approximately 0.2 nm. For the 2S mutant, we recognized increasing distance between Gln800 – Ala904 at the top of the bundle. At the same time, the distance between Ser815 – Gln888 at the bottom of the bundle was decreasing, pointing at closing the bottom of the bundle. Similar, yet more intensive, helix reorganization was observed for the 3S and 4S mutants especially during the last 2 ns of the pulling simulation. This suggests that these bundles are opening from the top, making H3 VBS more accessible for vinculin binding (Supplementary Figure S1).

To computationally investigate the stability of the mutated helix bundles in the absence of force, we compared the structural differences of wild-type R3 subdomain and the 4S mutant during subdomain refolding (Supplementary Figure S2). The refolding ability was measured on the Val808 – Gly896 vector located in the middle of the bundle. Although the 4S mutant showed increased Val808 – Gly896 distance during mechanical stretching, this distance returned to its original length within 7 ns of the relaxation simulation. This suggests that even the most heavily modified 4S mutant readily refolds to its equilibrated state and that the mechanically destabilizing mutations do not prevent refolding of the subdomain helix bundle.

In order to evaluate the effects of the mechanically destabilizing mutations on R3 subdomain thermal stability and secondary structure, we produced the corresponding His6-tagged talin fragments (amino acids 795 – 911) in *E*. *coli*. We observed robust protein expression and good solubility of the fragments. Because of surprisingly large differences in the electrophoretic mobility of the fragments in SDS-PAGE even in the presence of 8 M urea (Fig. 2h), we confirmed the molecular weights of the purified proteins by mass spectrometry. Size exclusion chromatography (SEC) was used to analyze solubility and size of the produced talin fragments. All studied fragments were found to be soluble and no protein aggregation was observed in SEC (Fig. 2i). However, 3S and 4S mutants showed faster penetration time, suggesting oligomeric structure. This was confirmed by inline right angle light scattering (RALS) analysis, where molecular weights for WT and 1S – 4S mutants were 12.7 kDa, 13.7 kDa, 14.2 kDa, 29.5 kDa and 27.6 kDa, respectively.

Circular dichroism (CD) spectroscopy was used to analyze folding and thermal stability of the purified talin fragments. In CD spectroscopy, wild-type talin R3 and 1S mutant showed closely matching spectra, while the 2S showed signs of decreased subdomain helicity. This decrease in the subdomain helicity was especially apparent for the 3S and 4S mutants. After deconvolution at 205–260 nm, the observed helical contents of WT, 1S, 2S, 3S and 4S were 79.7%, 77.4%, 51.2%, 25.8% and 26.4%, respectively (Fig. 2j). Similarly, in CD spectroscopy melting analysis (Fig. 2k) the 1S mutant was found to have a moderately decreased thermal stability (T_m_ 52.6 °C) as compared to the wild-type fragment (T_m_ 66.9 °C), while the 2S, 3S and 4S mutations resulted in a further decrease in T_m_ value (48.0 °C, 40.7 °C and 40.9 °C, respectively). These results suggest that that at least in the conditions used during the analysis and in the absence of the surrounding talin domains, extensive mutagenesis of the hydrophobic core of the R3 helix bundle reduces the helical content and may expose hydrophobic patches, leading to dimerization in the case of 3S and 4S. Some indications of such behavior were also observed in MD simulations, where the mutated site was found to bend away from its original position, causing torsion of the helix.

Overall, the biochemical analyses indicate that 1S mutant resembles WT in all the characteristics while having decreased stability. The 3S and 4S mutants have severely affected folding and represent significantly destabilized R3 subdomains. The 2S mutant appears an intermediate between 1S and 3S. Therefore, we were keen to evaluate how these mutants would affect cellular talin-mediated functions.

### Talin R3 subdomain destabilization induces talin accumulation into adhesions independently of the presence of talin R4 - R12 subdomains

**Figure 3:**
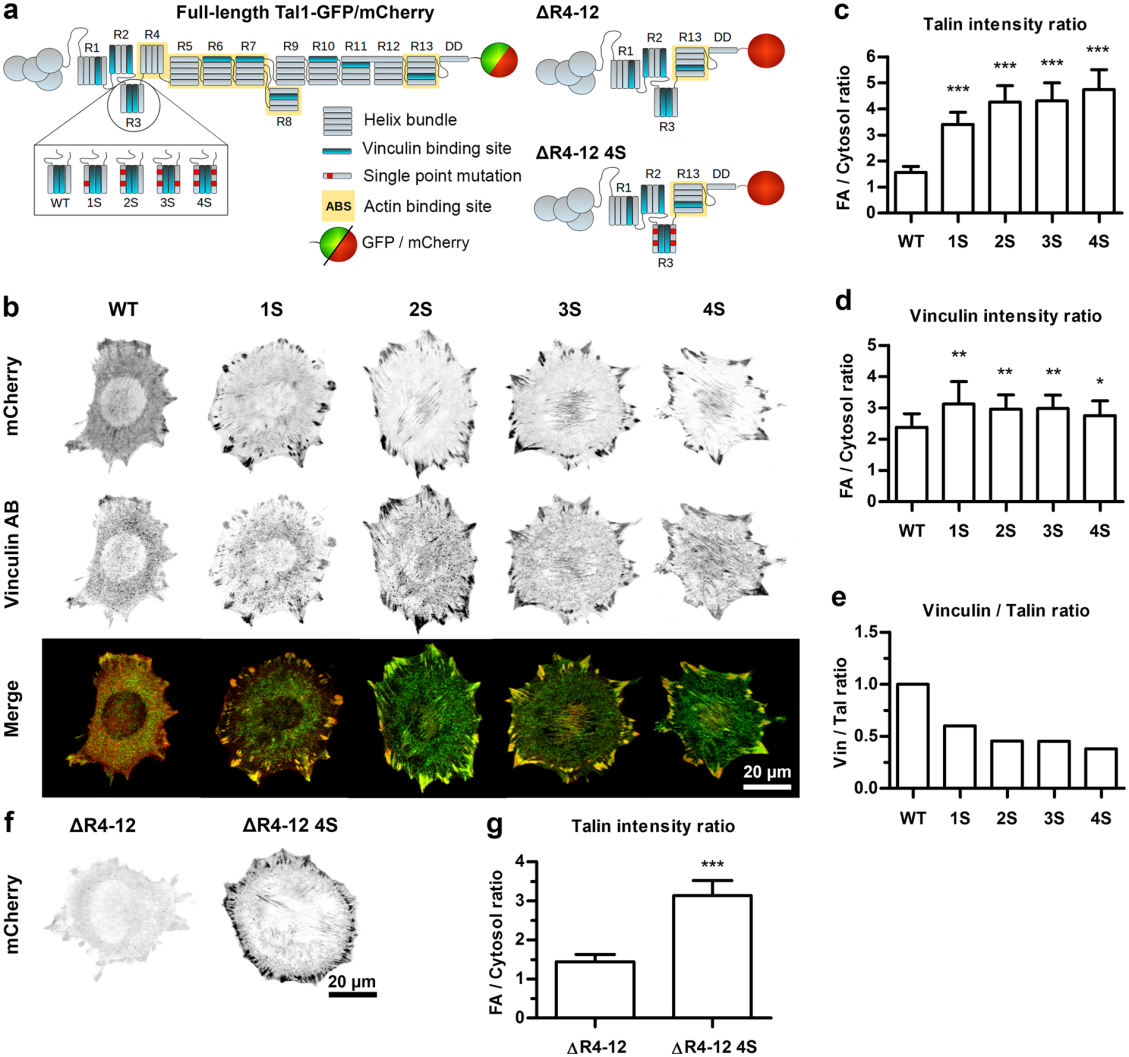
Talin-1 accumulation into cell-matrix adhesions in wild type MEF cells. (**a**) Schematic illustrations of full-length talin-1 constructs and truncated talin-1 constructs lacking rod subdomains R4-R12. The truncated talin ΔR4-12 construct does not contain any destabilizing mutations, while the ΔR4-12 4S construct contains all four point mutations presented in the Fig. 1a. (**b**) Representative images of wild type MEF cells expressing mCherry-tagged talin-1 proteins. In merged images, mCherry signal is shown in red and vinculin antibody staining as green. Colocalization of the two signals is indicated by yellow color. Scale bar is 20 µm. (**c**) Mean ratios of mCherry signal intensities measured from adhesion sites and cytosolic areas in wild type MEF cells expressing mCherry-tagged talin-1 mutant proteins. Error bars represent SD. n = 15 cells for each talin construct. The ratios measured for the talin-1 mutants were compared to mCherry-tagged wild type talin-1 by One-way ANOVA, ***p < 0.0001. (**d**) Mean adhesion/cytosolic signal ratios for vinculin antibody staining of cells expressing mCherry-tagged talin-1 forms. Error bars represent SD. n = 22, 20, 15, 15 and 17 cells for wild type talin-1 and 1S, 2S, 3S and 4S mutants, respectively. Comparison to cells expressing wild type talin-1 by one-way ANOVA. **p < 0.001, *p < 0.01. (**e**) Vinculin/talin-1 ratios for results in (**a**,**b**). (**f**) Representative images of wild type MEF cells expressing truncated mCherry-tagged talin-1 mutant proteins. Scale bar is 20 µm. (**g**) Mean ratios of mCherry signal intensities measured from adhesion sites and cytosolic areas in wild type MEF cells expressing truncated mCherry-tagged talin proteins. Error bars represent SD. n = 15 cells for each construct. Statistical analysis by two-tailed t-test. ***p < 0.0001.

The destabilizing talin mutations were introduced into full-length talin expression constructs to study their effects on talin and vinculin recruitment into cell-matrix adhesions. Wild-type mouse embryonic fibroblast (MEF) cells were transfected with the talin constructs, fixed and immunostained for vinculin. Based on captured microscope images, fluorescence intensity ratios between adhesions and cytosolic areas were determined to minimize the bias caused by cell-to-cell variance in the expression levels of the talin proteins. Interestingly, even a single point mutation (1S mutant) in the R3 subdomain was enough to significantly increase this ratio, indicating increased talin partitioning into cell-matrix adhesions (Fig. 3c). Further mutagenesis increased the ratio up to three-fold (for the 4S construct) as compared to the wild-type talin construct. Similar increase in the intensity ratio was also observed for vinculin staining (Fig. 3d). However, the fold increase in this ratio for vinculin was smaller than that observed for talin, resulting in a decrease in the vinculin/talin ratio for the destabilized talin mutants (Fig. 3e). Also the maximal increase in vinculin intensity ratio was already achieved by introducing one destabilizing point mutation (1S mutant). Consequently, the highest vinculin/talin ratio was observed for wild type talin-1 and the ratio gradually decreased when additional point mutations were introduced into the construct. The observed decrease in the vinculin/talin ratio likely resulted from vinculin binding to the abnormally active VBS in talin R3, which activates downstream signaling cascades, whilst decreasing cellular traction forces generation. This, in turn, decreased the force applied on individual talin and results in a decrease in the number of activated VBSs (overall, there are 11 VBSs in talin rod and only two of them are within R3). Similar increase in talin accumulation and decrease in vinculin/talin ratio was also observed in TLN1−/−TLN2−/− cells, confirming that the results are not dependent on the presence of endogenous talin proteins (Supplementary Figure S3c,e). To confirm similar expression levels and the expression of intact talin protein for all talin constructs, lysates of TLN1−/−TLN2−/− cells expressing the studied talin proteins were analyzed by using Western blot with anti-mCherry antibody (Supplementary Figure S3b).

In a previous study, a cryptic actin binding site (ABS2) in talin-1 rod R4-R8 subdomains was proposed to be a key factor in controlling the incorporation of talin into adhesion structures^15^. To rule out the possibility that the increased accumulation of the destabilized talin constructs would result from altered regulation of talin autoinhibition or talin ABS2, we also studied the 4S mutations in the context of a truncated talin-1 mutant lacking subdomains R4 - R12. This centrally truncated talin–1 (Tal1 ΔR4-12) accumulated only very weakly into cell-matrix adhesions, resulting in a low adhesion/cytosol intensity ratio and poorly visible adhesions (Fig. 3f). However, introduction of the four destabilizing mutations (Tal1 ΔR4-12 4S) into this construct caused a similar increase in the adhesion/cytosol intensity ratio as was seen with the full length talin (Fig. 3f,g), indicating that other talin rod subdomains are not required for the enhanced cell-matrix adhesion recruitment caused by the destabilizing mutations. This suggests that the increase in talin-1 adhesion localization was indeed dependent on the unfolding of the talin-1 R3 subdomain and not as a result from disturbed autoinhibition of talin-1 ABS2^15^. In addition, because both Tal1 ΔR4-12 and Tal1 ΔR4-12 4S lack the R9 subdomain needed in the talin autoinhibitory interaction between the talin-1 head F3 domain and the rod R9 subdomain^16, 17^, these results confirm that increased accumulation of destabilized full length talin is not caused by disrupted talin autoinhibition.

### Talin R3 subdomain destabilization affects traction force generation, talin and vinculin mobility in adhesions and the rate of cell migration

**Figure 4:**
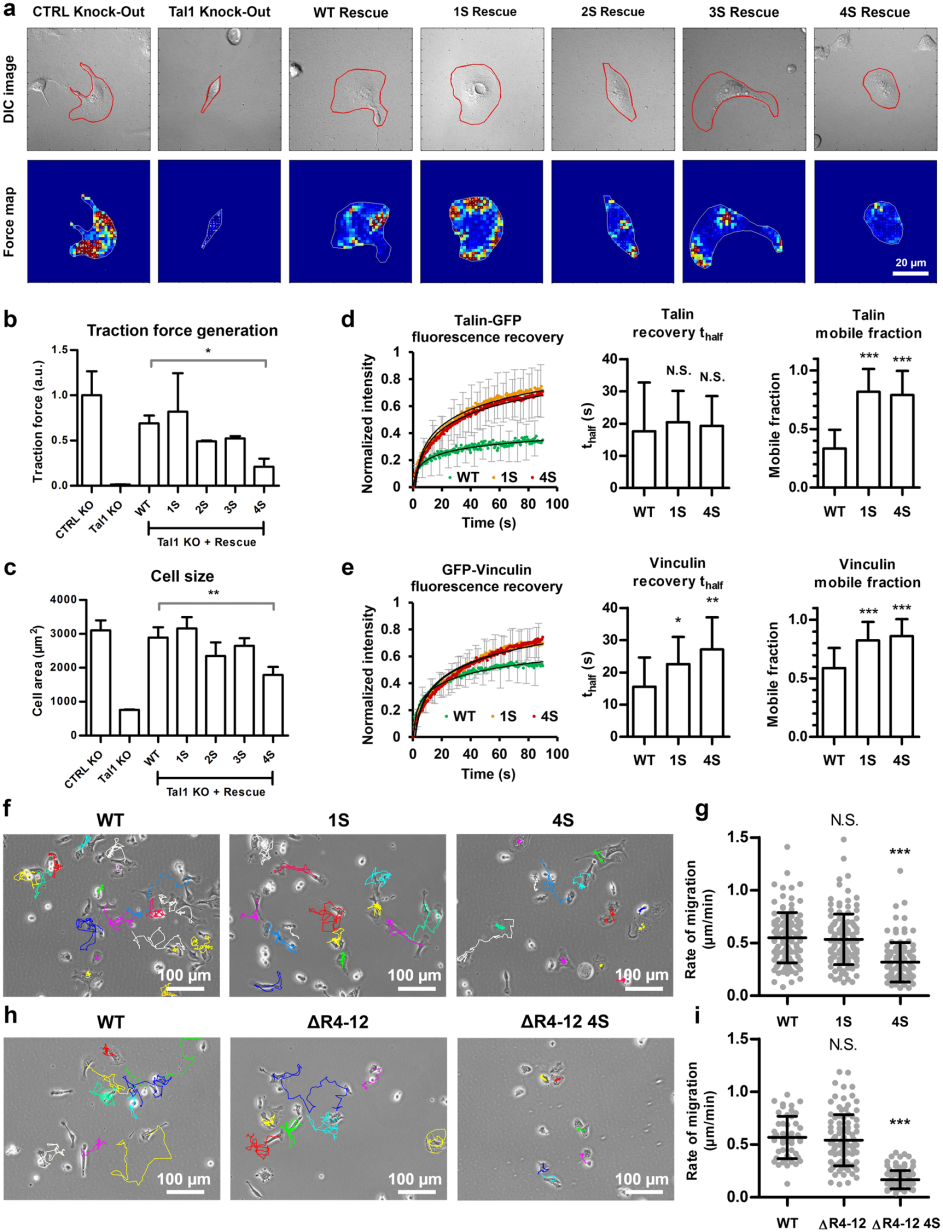
Talin destabilization decreased traction force generation and the rate of cell migration and increased talin and vinculin mobility in cell-matrix adhesions. (**a**) Representative traction force microscopy force maps and DIC images of talin-1 knock-out U2OS cells expressing talin-1 constructs. In the force maps, low local forces are illustrated by blue and green colors, while high local forces are illustrated by yellow and red colors. (**b**) Traction force analysis for talin-1 knock-out U2OS cells. Data are means ± SD. n = 5, 3, 4, 5, 3, 3, 4 for samples named in the graph legend from left to right. Comparison to cells expressing wild type talin-1 by one-way ANOVA and Tukey’s test. *p < 0.05, Talin-1 WT vs. Talin-1 1S, 2S or 3S were not statistically significant. (**c**) Cell area analysis for talin-1 knock-out U2OS cells. Data are means ± SD, n = 6, 3, 5, 4, 3, 3 and 4 cells for samples named in the graph legend from left to right. Comparison to cells expressing wild type talin-1 by one-way ANOVA. **p < 0.001, Talin-1 WT vs. Talin-1 1S, 2S or 3S were not statistically significant. (**d**) Talin-GFP FRAP recovery curve, intensity recovery half time and mobile fraction for wild type talin and destabilized 1S and 4S mutants. Average of 24 cells for each construct from two fully independent experiments. Error bars represent SD. Comparison to cells expressing wild type talin by one-way ANOVA. ***p < 0.0001. (**e**) GFP-vinculin FRAP recovery curve, intensity recovery half time and mobile fraction for cells expressing Talin-mCherry constructs. Average of 29, 24 or 25 cells for WT, 1S and 4S, respectively, from two fully independent experiments. Error bars represent SD. Comparison to cells expressing wild type talin by one-way ANOVA. **p < 0.001, *p < 0.01. (**f**,**h**) Representative 12-hour migration tracks for *TLN1*−/−*TLN2*−/− MEF cells expressing EGFP-tagged wild type talin-1 or full-length 1S and 4S talin mutants (**f**) or mCherry-tagged full-length talin and truncated talin forms (**h**). Scale bars are 100 µm (**g**,**i**) Quantification of migration rates for cells expressing full-length or truncated talin-1 constructs. Horizontal lines indicate mean ± SD. For each construct, the results were pooled from two fully independent experiments. n = 116, 122 and 111 cells for wild type talin-1 and full length 1S and 4S talin mutants (**g**). n = 49, 98 and 105 for wild type talin-1 and ΔR4-12 and ΔR4-12 4S mutants (**i**). Other samples were compared to cells expressing wild type talin-1 by one-way ANOVA and Tukey’s test. N.S. = Difference not statistically significant, ***p < 0.0001.

Cell-matrix adhesions are important signaling hubs for the regulation of actomyosin contractility and cellular traction force generation. In turn, forces acting on the adhesion components are in part regulating adhesion dynamics. To study the effects of talin destabilization on cellular traction force generation, local traction forces were determined by using fluorescent 0.2 µm beads embedded into polyacrylamide gel. Talin-1 in human osteosarcoma U2OS cells was ablated by CRISPR techniques as described by Qi *et al*.^18^. Talin-1 ablation resulted in a large decrease in the average cell area and in a complete loss of cellular traction forces (Fig. 4a–c). Re-expression of GFP-tagged wild-type talin-1 fully rescued cell spreading and largely rescued traction force generation. Re-expression of the destabilized talin proteins rescued cell spreading and traction force generation to a degree dependent on the mechanical stability of the expressed talin protein. Re-expression of the 1S construct fully rescued cell spreading and caused a small, statistically insignificant increase in cellular traction forces as compared to the cells expressing wild-type talin. It is important to notice that although the 1S mutant rescued the total traction force generation to the same level as wild-type talin, its increased accumulation into cell-matrix adhesions (Fig. 3c) most likely leads to a decrease in the forces applied to each individual talin molecule. Re-expression of 2S or 3S forms rescued both cell spreading and traction force generation to slightly lower levels than wild-type talin-1. Re-expression of the 4S form resulted in only a partial rescue of both cell spreading and traction force generation, indicating that drastic talin-1 R3 subdomain destabilization results in severe defects in cellular traction force generation and mechanosignaling.

Fluorescence recovery after photobleaching (FRAP) experiments were performed to analyze how the mechanically destabilizing mutations affect talin and vinculin turnover rates in cell-matrix adhesions. For talin and vinculin FRAP, *TLN1*−/−*TLN2*−/− cells were either transfected with Tal1-GFP or co-transfected with Tal1-mCherry and GFP-Vinculin, respectively, and imaged on a confocal microscope. Talin destabilization by 1S and 4S mutants increased the mobile fraction of talin localized to cell-matrix adhesions (mobile fractions of 0.33, 0.82 and 0.79 for WT, 1S and 4S, respectively), indicating increased mobility of the 1S and 4S mutants in cell-matrix adhesions (Fig. 4d). Co-expression of destabilized talin proteins also significantly increased the mobile fraction of GFP-vinculin, but the effect was not as large as it was for talin itself (mobile fractions of 0.59, 0.83 and 0.86 for WT, 1S and 4S, respectively) (Fig. 4e). Interestingly, co-expression of destabilized talin proteins also increased the fluorescence recovery half time of GFP-vinculin as compared to co-expression of wild type talin. This indicates that the exchange rate of GFP-vinculin was slower when it was co-expressed with destabilized talin proteins. This could be caused by slower refolding of the destabilized talin proteins, especially when under mechanical force.

To analyze the effects that talin destabilization has on the rate of cell migration, *TLN1*−/−*TLN2*−/− MEF cells were transfected with wild type and destabilized talin constructs and cultured for 12 h on a fibronectin coated surface. Introduction of a single destabilizing mutation (1S mutant) into the talin-1 R3 domain did not significantly affect the observed rate of migration of talin-1 transfected *TLN1*−/−*TLN2*−/− MEF cells (Fig. 4f,g). However, introduction of all four mutations (4S mutant) resulted in a significant decrease in the rate of migration, although it did not completely block migration. To analyze adhesion maturation and disassembly in these cells in detail, we used live-cell fluorescence imaging with cells expressing either GFP-tagged WT talin-1 or talin-1 4S mutant. Although talin-1 R3 subdomain destabilization caused a striking increase in its accumulation into cell-matrix adhesions, no other obvious differences in adhesion formation and disassembly were observed during a 120 min live cell imaging (Supplementary Movie S5). Surprisingly, deletion of the talin-1 rod R4 - R12 subdomains did not significantly affect the rate of cell migration (Fig. 4i). *TLN1*−/−*TLN2*−/− MEF cells expressing the truncated talin ΔR4-12 protein were still able to polarize and the rate of migration was comparable to that of cells expressing wild type talin-1. On the contrary, cells expressing the 4S-destabilized talin-1 ΔR4-12 construct were able to spread, but their ability to migrate was fully blocked (Fig. 4i). Apart from small movement resulting from seemingly random detachment of lamellipodium on one side of the cell, cells expressing this construct were unable to migrate. This suggests that the correct mechanical stability of the R3 subdomain is indispensable for cell migration in the absence of rod subdomains 4-12, but not in the context of full length talin.

### Talin-1 R3 subdomain destabilization affects ECM sensing and talin colocalization with β3 and β1 integrin subtypes

**Figure 5:**
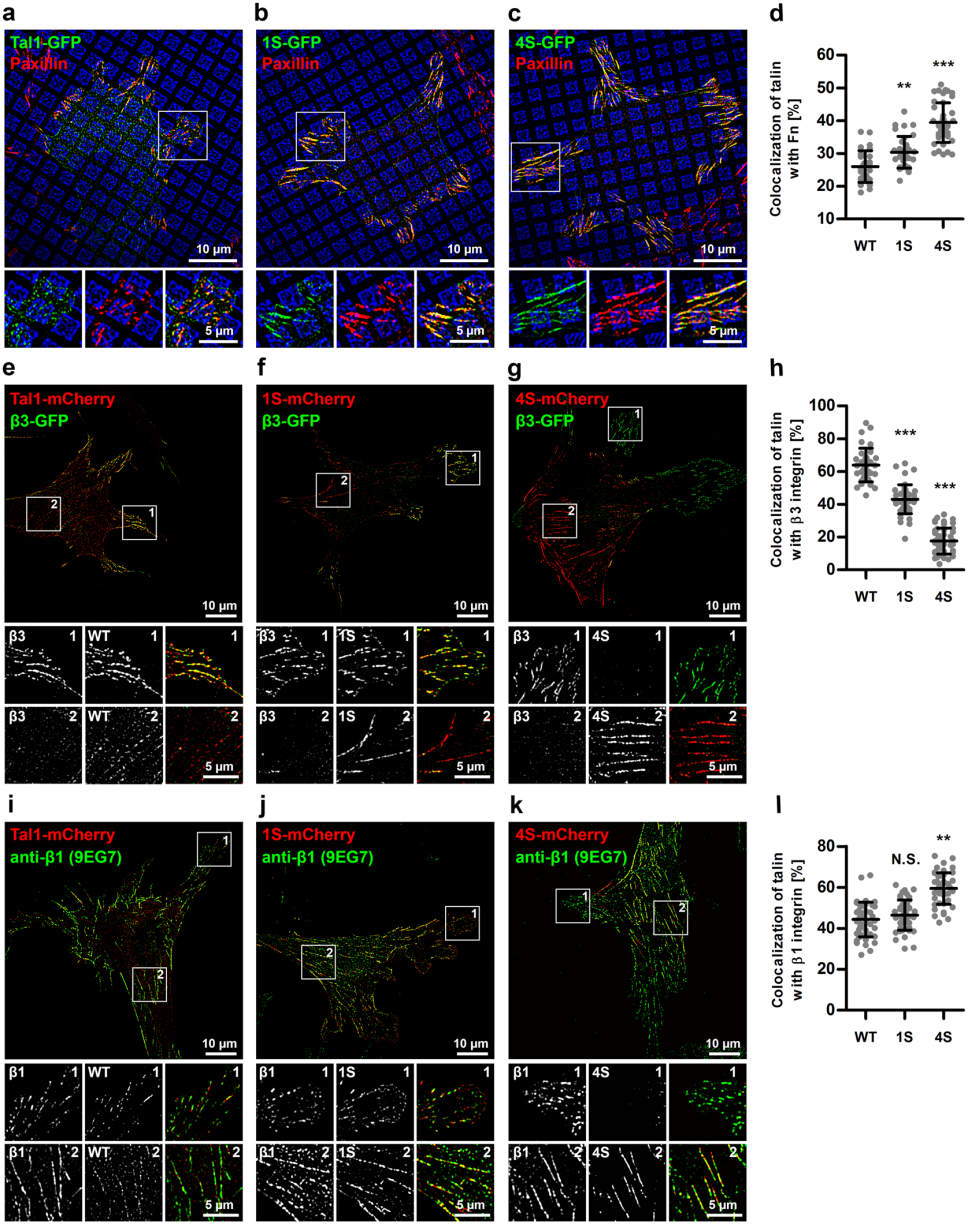
Analysis of talin recruitment to cell-matrix adhesions on Fn/Vn patterned substrates and talin colocalization with β3 and β1 integrin subtypes. (**a**–**c**) Representative SIM images of transfected wild type MEF cells cultured on micropatterned substrates with 56% of area coated with fibronectin (blue squares) and 44% of area coated with vitronectin (black background). Talin1-GFP fluorescence signal is shown as green and paxillin antibody staining used as a reference for cell-matrix adhesion localization is shown as red. (**d**) Quantification of talin recruitment to cell-matrix adhesions on Fn coated areas. Data were pooled from three fully independent experiments, n = 30, 34 and 43 for cells expressing wild type talin-1, 1S mutant or 4S mutant, respectively. Mean ± SD. Statistical analysis by one-way ANOVA and Tukey’s test. **p < 0.001, ***p < 0.0001. (**e**–**g**) Representative SIM images of cells cotransfected with talin mutants and β3-GFP and cultured on fibronectin-coated substrate. Notice how talin 4S mostly localized to central adhesions, while β3 integrin only localized to peripheral adhesions. (**h**) Quantification of talin colocalization with β3-GFP. n = 36, 44 and 47 cells for WT, 1S and 4S, respectively, pooled from three fully independent experiments. Mean ± SD. Statistical analysis by one-way ANOVA and Tukey’s test. ***p < 0.0001. (**i**–**k**) Representative SIM images of cells transfected with talin mutants and immunostained with 9EG7 antibody for activated β1 integrin. (**l**) Quantification of talin colocalization with β3-GFP. n = 46, 48 and 47 cells for WT, 1S and 4S, respectively, pooled from three fully independent experiments. Mean ± SD. Statistical analysis by one-way ANOVA and Tukey’s test. **p < 0.001.

The structure of a cell-matrix adhesion is tightly linked to its function and it is constantly changing as a response to intracellular and extracellular signals. Changes in the structure and function of the intracellular adhesion complex are also reflected on the extracellular side of the plasma membrane, as demonstrated by the different integrins clustered in different adhesion subtypes^19^. To study the nature of cell-matrix adhesions induced by the destabilized mutants, wild type MEF cells expressing GFP-tagged wild type talin-1 or destabilized 1S or 4S mutants were cultured on coverslips micropatterned with fibronectin and vitronectin spatially separated from each other on a subcellular resolution, as described previously in Pinon *et al*.^20^. Imaging with superresolution microscopy (SIM) revealed that in the cells expressing wild type talin-1 adhesions were shorter and more punctuated, as compared to the long, streak-like adhesions seen in the cells expressing 1S and 4S mutants (Fig. 5). To minimize the bias caused by unspecific cytosolic fluorescence signal from the talin-1-GFP constructs, analysis of adhesion localization was limited to areas within a mask based on the paxillin antibody staining (Supplementary Figure S6). Adhesions associated with wild type talin-1 strongly favored vitronectin over fibronectin, with only 26% of adhesion area on fibronectin coated surfaces. Talin-1 destabilization caused a decrease in this preference, as indicated by 31% and 40% of adhesions on fibronectin coated areas for cells expressing the 1S and 4S mutants, respectively.

To study the interactions of integrin subtypes with the destabilized talin proteins, we coexpressed WT, 1S or 4S talins with GFP-tagged β3-integrin on glass coverslips coated with fibronectin (Fig. 5e–l). This analysis revealed that the localization of the talin forms varied so that WT and 1S preferentially accumulated into the cell periphery (Fig. 5e,f), while 4S was mostly found in central adhesions within the cell body (Fig. 5g). The peripheral adhesions containing WT and 1S talins were found to be strongly associated with β3 integrin (Fig. 5e,f,h), while the central adhesions rich in 4S contained only negligible amounts of β3 integrin (Fig. 5g,h). We also studied the colocalization of talin forms with β1 integrin using 9EG7 antibody specific for activated β1-integrin (Fig. 5i–k). Talin 4S mutant showed increased colocalization with β1-integrin as compared to WT, indicating that the stability of talin rod domain can regulate the usage of β-integrin subtypes. These changes in the preference for different ECM proteins and the usage of different β-integrin subtypes suggest that talin-1 destabilization does not only affect adhesion dynamics, but also the protein composition of cell-matrix adhesions and adhesion segregation^19^.

## Discussion

Talin is a central adhesion scaffold protein and it is intensively involved in integrin-mediated processes. It contains confirmed or putative binding sites for at least ten other adhesion proteins, thus regulating their recruitment and activation. The mechanical properties of the talin rod domain have been proposed to be an important factor in the regulation of mechanosensing and mechanosignalling. The forces needed to unfold the talin rod subdomains *in vitro* cover a wide range, from 5–10 pN for talin R3 to 25–40 pN for R9^9, 10^. The working hypothesis of this study was that the mechanical stabilities of the rod subdomains are optimized for cellular mechanotransduction and that the gradual unfolding of talin rod subdomain allows cells to measure small local changes in cellular forces at a high resolution. Despite of the vast amount of talin-focused research, mechanically altered talins have not been thoroughly characterized in previous studies. In particular, the effects of talin destabilization on the regulation of cellular processes are unknown.

Talin-1 R3 subdomain is in many ways unique among the talin-1 rod subdomains. It has binding sites for both vinculin and RIAM and its mechanical stability is the lowest of all rod subdomains^8, 13, 14^. Because of the low mechanical stability of the R3 subdomain, it may have a key role in the mechanotransduction of low magnitude forces during the maturation of nascent adhesions. If talin acts as a mechanosensor, any alteration to its mechanical stability is likely to affect adhesion dynamics and cellular mechanosensing. Accordingly, in a previous study by Elosegui-Artola *et al*., the expression of a mechanically stabilized talin R3 mutant called IVVI was shown to increase the threshold of the substrate rigidity needed for triggering cellular traction force generation and YAP signaling^13^. The likely reason for this increase in the threshold rigidity is that on softer substrates, the tensional forces are not high enough for force-induced unfolding of the stabilized R3 subdomain, which prevents vinculin recruitment to R3 subdomain and downstream signaling. In line with these findings, the destabilizing mutations described in the present study were found to make talin more sensitive for the mechanotransduction of low magnitude forces, as indicated by the observed decreases in both cell migration rate and traction force generation.

**Figure 6:**
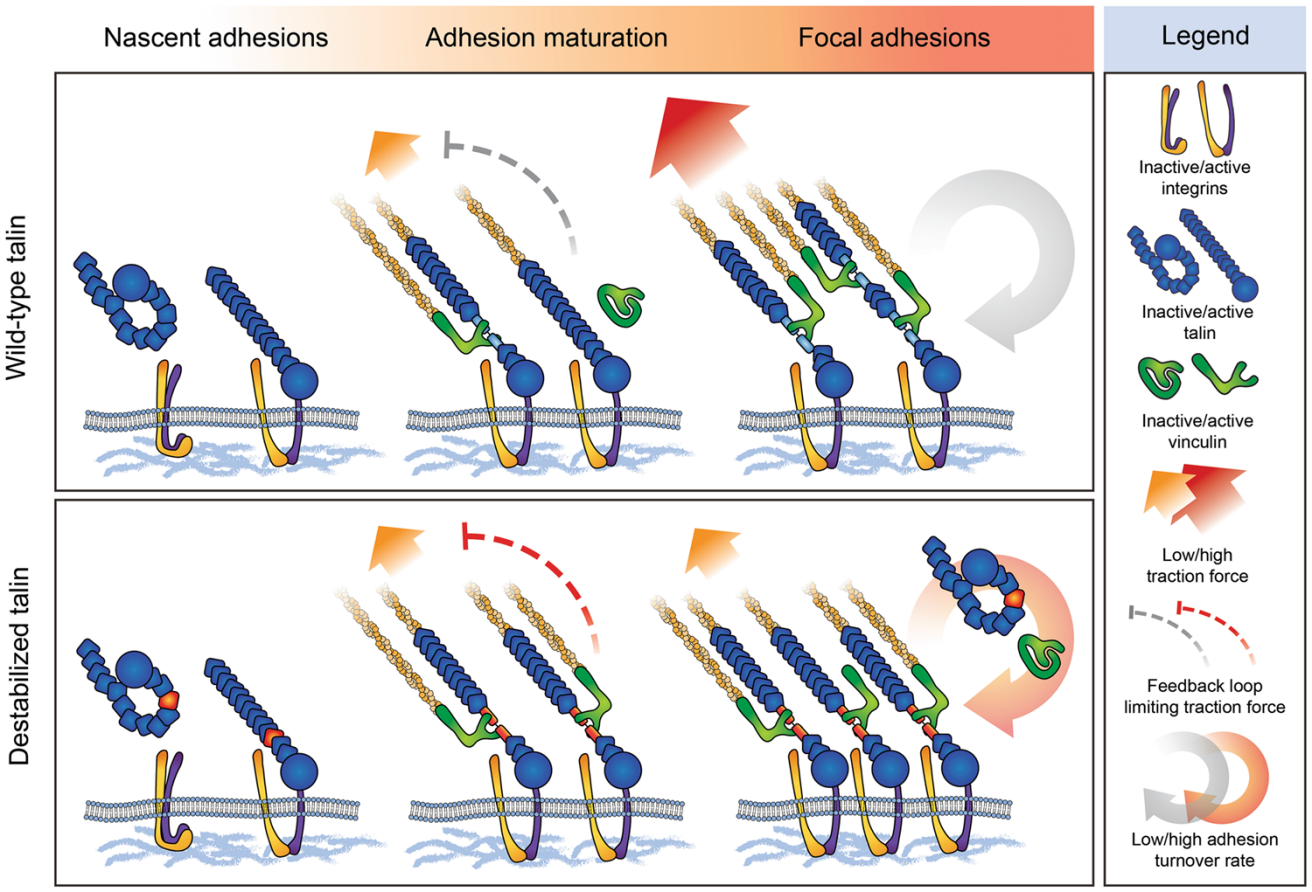
Model for decreased traction force generation and increased talin accumulation after talin rod domain destabilization. Talin is recruited into **nascent adhesions** in its autoinhibited conformation and in the absence of mechanical load. Other adhesion proteins participating in the recruitment of talin are omitted from the schematic representation for simplicity. During **adhesion maturation**, mechanical forces transmitted through the talin rod domain promote unfolding of the rod subdomains. Subdomain unfolding exposes cryptic VBSs and allows vinculin binding. Destabilized talin R3 subdomains unfold at lower force, facilitating subdomain unfolding and vinculin binding. Talin rod subdomain unfolding and vinculin binding initiate negative feedback loops that limit cellular traction force generation and adhesion growth. Decreased unfolding force of the destabilized talin rod R3 subdomain results in the activation of this negative feedback also in adhesions transmitting low magnitude forces, which decreases overall traction force generation. In mature **focal adhesions**, talin rod subdomains unfold at their characteristic threshold forces, creating a heterogeneous pool of talin molecules with rod subdomains unfolded to various degrees. Together with the decrease in cellular traction force generation, the intense accumulation of destabilized talin proteins into focal adhesions results in a large decrease in the force transmitted by an individual talin protein, which increases the mobility of talin proteins within focal adhesions. Decreased traction force generation also affects the unfolding of other talin rod domain bundles, which results in a decrease in the vinculin/talin ratio in these adhesions.

Although talin is recruited into nascent adhesions independently of vinculin or transmitted mechanical force, both mechanical stretching of the talin rod domain and recruitment of vinculin are needed for stabilizing talin into cell-matrix adhesions^21, 22^. Interestingly, we found that destabilization of the talin R3 subdomain strongly promoted talin accumulation into cell-matrix adhesions (Fig. 3c). Although slightly increased adhesion accumulation was also observed for vinculin, the vinculin/talin ratio was decreased in cells expressing the destabilized talin mutants. This decrease in vinculin/talin-ratio was accompanied by a decrease in the magnitude of cellular traction forces (Fig. 4b). This likely results from facilitated unfolding of the talin R3 subdomain, especially in adhesions transmitting low-magnitude forces. This leads to the exposure of cryptic VBSs in adhesions where the level of mechanical tensions would not normally be sufficient for talin subdomain unfolding (Fig. 6). Such talin unfolding and the resulting vinculin binding possibly trigger downstream signaling cascades that eventually downregulate cellular traction force generation. Decreased traction force generation, in turn, prevents further unfolding of talin rod subdomains and results in a decreased vinculin/talin ratio, as seen for the cells expressing destabilized talin mutants (Fig. 3e).

In the FRAP experiments, talin destabilization vastly increased the mobile fraction of talin localized into cell-matrix adhesions (Fig. 4d). Slightly surprisingly, this suggests that the large and protein-rich adhesions organized around mechanically destabilized talin proteins have a highly dynamic structure. Thus the majority of the intergrin-bound talin proteins are being constantly exchanged with the cytosolic pool of talin proteins. This finding is in line with the decreased traction force generation observed in this study and highlights the importance of mechanical force as a regulator of adhesion turnover^21^. Similarly to the increased mobile fraction observed for destabilized talin proteins, the mobile fraction of vinculin was increased after talin mutagenesis. However, at the same time we also observed increased FRAP half-recovery time for vinculin. This indicates that despite of the increased mobile fraction, vinculin shows slower initial fluorescence recovery after talin destabilization. This decrease in the vinculin turnover rate may be caused by decelerated dissociation of vinculin-talin complex due to slower refolding of talin rod subdomain after its release from integrin cytoplasmic domain. This is supported by the biochemical analysis of talin R3 mutants, where we found 3S and 4S being rather unstable and poorly folded as isolated domains when compared to WT, 1S and 2S (Fig. 2). Based on these findings, it seems clear that talin destabilization causes defects in cellular mechanosignaling and eventually lowers the force applied on an individual talin protein. This results in increased adhesion protein dynamics and affects the vinculin/talin ratio within cell-matrix adhesions (Fig. 6). The molecular feedback mechanism linking the talin-vinculin interaction to the regulation of applied mechanical load is currently unknown and it may involve several different proteins.

In addition to the increased talin mobile fraction and accumulation into cell-matrix adhesions, R3 destabilization also affected the size, shape and protein composition of cell-matrix adhesions. While wild type talin1-GFP localized mainly into short and roughly circular adhesions at the cell periphery, destabilized talin mutants localized into long, streak-like adhesions that were often found also in the central parts of the cell (Figs 3b and 5). To get a better understanding of the nature of the adhesions formed around the destabilized talin proteins, we analyzed the preference of modified talin proteins to accumulate on fibronectin or vitronectin coated areas. Interestingly, talin destabilization was found to strongly promote adhesion formation on fibronectin (Fig. 5d). In accordance with this finding, we also observed changes in the usage of different β-integrin subtypes after talin rod domain destabilization. These findings indicate that talin rod domain stability is indeed regulating the structure and function of cell-matrix adhesions at the levels of substrate sensing and integrin activation. However, the mechanism that links talin rod domain stability and differential activation of integrin subtypes remains to be found.

In the migration analysis, we observed decreased migration rates for cells expressing destabilized full-length talin-1. Together with the observed accumulation of destabilized talin proteins into cell-matrix adhesions, these results suggest that regulation of adhesion dynamics is controlled by the mechanical stability of talin. These findings are in line with a previous study by Carisey *et al*., where mutations releasing the vinculin head-tail autoinhibition were shown to lock talin in cell-matrix adhesions, resulting in slower adhesion turnover and decreased cell polarization and migration^21^. In both cases, disturbances in the tight spatial and temporal control of talin-conformation regulated interactions negatively affected the ability of cells to polarize and migrate. It is possible that the defects in cell polarization and migration result from the failure of cells to probe local cellular tension because of the facilitated formation of the talin-vinculin interaction under low force. In this study, we found that neither talin-1 subdomain R3 destabilization nor deletion of the R4-R12 subdomains alone are sufficient to block cell migration. However, together they result in a completely static cell phenotype in talin-null cells (Fig. 4i). The impaired migration of these cells may result from the lack of cryptic and mechano-activatable vinculin binding sites in the talin-1 mutant that is both truncated and destabilized. It is possible that in the context of full-length talin, the defects in mechanosensing caused by the R3 subdomain destabilization can be partly compensated by the presence of other cryptic and mechano-activable vinculin binding sites in the other rod subdomains. We propose that unfolding of mechanosensitive talin-1 rod subdomains results in local differences in the level of talin-initiated signaling in the cell. These local differences in signaling activity are a prerequisite for cell polarization and migration and the lack of them after both truncating and destabilizing talin rod domain would explain the severely impaired cell migration seen for the cells expressing this construct.

Talin R3 subdomain does not only contain two cryptic vinculin binding sites, but it also has a mutually exclusive binding site for a Rap1 effector protein RIAM. RIAM is thought to be an important regulator of talin recruitment to the plasma membrane and it is present mainly in nascent adhesions, while vinculin binding is needed for adhesion reinforcement and locking talin into mature focal adhesions^23, 24^. Force-induced unfolding of the R3 subdomain likely acts as a switch releasing RIAM and allowing vinculin binding during adhesion maturation^14^. In the current study, we found that talin R3 subdomain destabilization increased talin and vinculin accumulation into cell-matrix adhesions, which likely indicates a decrease in the threshold force at which the transition from the RIAM-binding conformation to the vinculin-binding conformation occurs. This decrease in the threshold force facilitates the release of RIAM and simultaneously promotes vinculin binding. However, the mutations presented in this study are unlikely to directly affect the formation of the talin-RIAM complex, because they do not directly target the RIAM binding site in talin R3 helices H2 and H3. Moreover, RIAM binding to talin presumably occurs in the absence of mechanical load applied to talin^14^. To study this experimentally, we coexpressed talin mutants and RIAM-GFP and did not observe significant differences in RIAM localization between wild-type and destabilized talin proteins (Supplementary Figure S4). In addition to vinculin and RIAM binding, the R3 subdomain has been suggested to participate in the regulation of actin binding site 2 (ABS2), located in the talin rod subdomains R4 and R8^15^. Interestingly, Atherton *et al*. found that deletion of the R2 and R3 subdomains resulted in the formation of larger and more stable cell-matrix adhesions. Their experiments using talin mutants with reduced actin binding suggested that the R2-R3 region may have a role in the regulation of talin ABS2, which is located in the rod subdomains R4 and R8. Although the destabilizing mutations described in the present study could affect the regulation of ABS2 in an indirect way by promoting forced unfolding of the R3 subdomain, our experiments with the talin ΔR4-12 4S mutant show that the increased talin accumulation into adhesions is independent of the presence of ABS2 (Fig. 3g). Therefore, it seems that the effects caused by talin R3 subdomain destabilization cannot be explained by the released autoinhibition of talin ABS2.

Our study highlights the importance of talin rod domain mechanical stability as a key factor in cellular mechanosensing. We demonstrate that the modulation of the mechanical stability of an individual talin rod subdomain affected a wide range of cellular processes dependent on mechanical signals and cellular mechanosensing. Our results suggest that talin acts as a mechanosensor together with vinculin and is responsible for controlling adhesion turnover, ECM sensing and consequently traction force generation and cell migration.

### Experimental procedures

#### Sequence analysis and mutation design

Multiple sequence alignment (Clustal Omega^25^) and conservation analysis for talin-1 R3 domain was performed in order to identify candidates for mutation design (Fig. 1c). The alignment was visualized and analyzed with Jalview^26^. PyMOL, Molecular graphics system, version 1.4.1^27^. Was used for visualization, target residue identification and following mutagenesis of four talin-1 R3 residues.

#### Molecular dynamics (MD) and steered molecular dynamics (SMD)

Gromacs version 4.6.7^28^. On Sisu supercomputer, CSC, Espoo, Finland was used for all MD and SMD simulations. All models were built with explicit TIP3P water model in 0.15 M KCl neutralized solution and placed into a 10 * 10 * 30 nm rectangular box. Each molecule was oriented in the water box so that the d3 vector (vector between V808 CA and G896 CA) was parallel to z-axis; i.e. the pulling direction was perpendicular to the H1-H4 orientation (Fig. 1, Supplementary Figure S1). The system was minimized with 100,000 steps and equilibrated for 10 ns before applying pulling force. Equilibration was performed with 2 fs step size at NTP conditions with v-rescale thermostat at 310 K for protein and solution applied separately and with the Berendsen barostat at 1 bar at isotropic setting. Simulations for domain stability assessment were performed at equilibration conditions for 20 ns. Simulations for domain refolding were performed at equilibration conditions for 20 ns starting from the structure coordinates after 10 ns pulling. Equilibration trajectory was recorded at 100 frames/ns for each analysis.

Pulling simulations were prepared to pull N-terminal helix H1 and C-terminal helix H4 apart in order to follow domain dissociation. Helices were pulled by 4 points in H4 at 150 pN constant force in the z-axis direction. In more detail, the alpha atoms of residues in helix 1 (H1; Q800, T804, V808, S815) were fixed, whilst alpha atoms of residues in helix 4 (H4; Q888, G896, A900 and A904) were pulled with constant force. The use of relatively high force is justified by the limitations in computing resources and based on previous studies suggesting predictive power for SMD simulations carried out using forces higher than observed in nature^6, 10^. Pulling simulations were performed with 2 fs step size for 10 ns and recorded at 500 frames/ns. Berendsen thermostat at 310 K was applied for protein and solution separately while Berendsen barostat at 1 bar was used at semiisotropic setting (pressure control was applied in xy coordinates, while pulling was performed in z-axis direction). This setup was selected based on extensive optimization of the parameters (von Essen *et al*., unpublished data). Equilibration was performed in one simulation experiment for each molecule. Stability and refolding simulations were simulated for wild type R3 subdomain and 4S mutant in one experiment. Finally, pulling simulations were repeated 5 times for each structure. All trajectories were analyzed with VMD^29^ at 100 frames/ns.

### Protein expression and purification

DNA fragments corresponding to the talin-1 residues 795 – 911 were subcloned into a modified pHis vector to create a construct with N-terminal His6-tag separated from the talin fragment by an 11 residue linker (SSSGPSASGTG). The expression of His6-tagged talin rod domain R3 protein in *Escherichia coli* BL21 Star cells (Invitrogen, USA) was induced by 1 mM IPTG for 5 h at 37 °C. Cell pellets were lysed by homogenization (Emulsiflex C3; Avestin, Inc., Germany) into pH 7.2 sodium phosphate buffer containing 1 M NaCl and 20 mM imidazole. After centrifugation, lysates were loaded into affinity column (HisTrap FF; GE Healthcare, UK) using a liquid chromatography system (Äkta purifier; GE Healthcare, UK). Proteins were eluted with a linear imidazole gradient 0–500 mM. Eluted fractions were run on SDS-PAGE gel. Samples purity were checked on the gels (>90%) and fractions containing proteins were dialyzed by stepwise decrease in imidazole concentration into 50 mM sodium phosphate buffer containing 150 mM NaCl pH 7.2. Dialyzed samples were again analyzed by SDS-PAGE and the concentrations detected by NanoDrop (Thermo Fisher Scientific, USA). Urea-PAGE gel was made in the same standard procedure as for SDS-PAGE gels, except 8 M urea was added. Samples were boiled in the sample buffer for 10 min at 100 °C. After cooling to room temperature, 8 M urea was added to the sample. Electrophoresis was performed at +4 °C, 100 V, for 2–3 hours. Protein concentration was determined by measuring A280 using NanoDrop. 1 mM EDTA and 1 mM DTT were added and protein samples were stored at +4 °C.

### Size exclusion chromatography

Analysis was performed using a liquid chromatography instrument (CBM-20A, Shimadzu Corporation, Kyoto, Japan) equipped with autosampler (SIL-20A), UV-VIS (SPD-20A) and Malvern Zetasizer μV SLS/DLS detector (Malvern Instruments Ltd, Worcestershire, UK). Data were processed using Lab Solution Version 5.51 (Shimadzu Corporation) and OmniSec 4.7 (Malvern Instruments) softwares. A sample of the protein (200 μg) was injected on a Superdex 200 10/300 GL column (GE Healthcare, Uppsala, Sweden) equilibrated with 50 mM NaH_3_PO_4_, 150 mM NaCl pH 7.2 buffer. Runs were performed with flow rate of 0.5 ml/min at 20 °C using a thermostated cabin. The molecular weight of the talin forms were determined using static light-scattering intensity (SLS) and the light scattering detector was calibrated using the monomeric peak of BSA.

### Talin fragment CD spectroscopy

CD spectra were recorded on a Chirascan instrument (Applied Photophysics, UK). Spectra were recorded between 200 and 280 nm with sampling points every 1 nm. Three scans were recorded and baseline spectra were subtracted from each spectrum. Quartz cuvettes with a 0.1-cm pathlength were used. Data was processed using Chirascan Pro-Data Viewer (Applied Photophysics, UK), CDNN^30^ written by Gerald Böhm (Martin-Luther Universität Halle-Wittenberg) and Microsoft Excel. The direct CD measurements (θ; mdeg) were converted into mean residue molar ellipticity ([θ]_MR_) by Pro-Data Viewer. In thermal unfolding experiment a 2 °C step size at 1 °C/min ramp rate with ± 0.2 °C tolerance was used. The melting temperature was analyzed with Global3 (Applied Photophysics).

### Mass spectrometry

The purified talin R3 fragments were analyzed by MALDI-TOF instrument (Ultraflex TOF/TOF, Bruker-Daltonics, Germany). A 10 µl aliquot of the protein solution was concentrated using a Millipore µ-C4 ZipTip pipette tip and mixed with a sinapic acid (Sigma Aldrich, USA) matrix. Single and double-charged peaks were detected without signs of significant impurities. The measured masses determined according to most intensive peaks were 14189.7, 14166.6, 14144.7, 14113.8 and 14088.2 (+1) and 7093.8, 7080.9, 7071.5, 7055.6 and 7041.8 (+2) for wild-type talin fragment and 1S, 2S, 3S and 4S mutants, respectively. The determined masses are reasonable when compared to the theoretical molecular weights calculated by Protparam.

### Cell lines and cell culture methods

The wild type MEF cell line was a kind gift from Dr. Wolfgang Ziegler and has been previously described by Xu & Baribault, 1998^31^. The *TLN1*−/−*TLN2*−/− MEF cell line has been previously described by Theodosiou *et al*., 2016^32^. Both cell lines were maintained in high-glucose DMEM supplemented with 10% FBS and 1% GlutaMax (Thermo Fisher Scientific, USA). A 37 °C incubator with 5% CO_2_ was used for culturing both cell lines.

### Expression constructs and transfection

C-terminally EGFP- or mCherry-tagged full-length mouse talin-1 expression constructs were created by subcloning talin-1 (1-2541) and EGFP or mCherry DNA fragments into a modified pEGFP-C1 vector backbone (Clontech, USA). Destabilizing point mutations were created with overlapping mutagenesis primers and silent restriction sites in the talin sequence. Centrally truncated and C-terminally mCherry-tagged talin ΔR4-12 and ΔR4-12 4S constructs were created by subcloning mouse talin-1 fragments corresponding to amino acids 1–913 and 2296–2541 into a modified pEGFP-C1 based backbone. All DNA constructs were authenticated by sequencing.

Wild type and *TLN1*−/−*TLN2*−/− mouse embryonal fibroblast (MEF) cells were transfected with Neon transfection system (Thermo Fisher Scientific, USA). For all constructs, 5 µg of plasmid DNA was used per 10^6^ cells. The following electroporation parameters were used for wild type and talin deficient MEF cells: 1350 V, 30 ms, 1 pulse and 1400 v, 30 ms, 1 pulse, respectively.

### Immunostaining and imaging of fixed cells

Zeiss high-performance 170 µm thick coverslips were coated with 25 µg/ml human fibronectin (Fn) for 1 hour at 37 °C and washed two times with PBS. Wild type MEF cells were transfected with talin constructs and allowed to recover for 24 h. The cells were trypsinized and plated at low confluency on Fn coated coverslips for 120 min, after which media was aspirated from the wells and the cells were fixed with 4% PFA in PBS (pH 7.4) for 15 min at RT. Fixed cells were washed two times with PBS and permeabilized with 0.2% Triton-X100 in PBS for 5 min at RT. Nonspecific antibody binding was blocked by incubating the samples in 5% FCS, 1% BSA and 0.05% Triton-X100 in PBS for 30 min at RT. Monoclonal anti-vinculin antibody (Clone hVIN-1, V9131, Sigma-Aldrich, USA) was diluted 1:400 in the blocking buffer and incubated on the coverslips for 1 h at RT. Coverslips were washed with PBS (3 × 5 min) before incubation with AlexaFluor-488 conjugated anti-mouse secondary antibody (A21202, Life Technologies, USA) diluted 1:250 in the blocking buffer. Immunostained coverslips were washed with PBS (3 × 10 min) and stored at +4 °C.

Fixed and immunostained samples were imaged with Nikon Eclipse Ti inverted microscope (Nikon Instruments, Japan) equipped with CFI Plan Apo VC 60x/1.40 Oil immersion objective (Nikon instruments, Japan), Yokogawa CSU10 spinning disk confocal unit (Yokogava, Japan) and Andor NEO sCMOS camera (Andor Technology, UK). 488 nm and 651 nm DPSS lasers were used for exciting AlexaFluor-488 and mCherry fluorophores, respectively. The coverslips were mounted to the microscope stage with a detachable steel chamber and kept immersed in PBS during imaging. All imaging parameters were kept constant for all samples to allow quantitative image analysis.

### FRAP analysis for talin and vinculin

For FRAP analysis of talin mutants, *TLN1*−/−*TLN2*−/− cells were transfected with C-terminally GFP-tagged talin constructs. For FRAP analysis of vinculin, cells were co-transfected with C-terminally mCherry-tagged talin constructs and N-terminally GFP-tagged full-length vinculin. Transfected cells were allowed to recover for 24 h and plated on fibronectin coated (25 µg/ml) glass-bottom dishes 2 h before imaging. Samples were allowed to further equilibrate at the microscope stage for 30 min before imaging was started. Zeiss Cell Observer. Z1 microscope equipped with LSM780 confocal unit, 37 °C/5% CO_2_ incubator and 63x/1.4 Oil immersion objective was used for imaging. Circular regions with a diameter of 2.6 µm were photobleached with 488 nm argon laser operated at a high intensity. Only one region in each cell was selected to be bleached. Confocal microscope images were captured at 500 ms intervals for 5s before photobleaching and for 90 s after photobleaching. Data was collected from to fully independent experiments using the same bleaching and imaging parameters for all samples. Fluorescence recovery was analyzed by using equation F = [B(t)/B(t < 0)]/[Cell(t)/Cell(t < 0)], where B(t < 0) and Cell(t < 0) are the average fluorescence intensities of the of bleached area and the entire cell, respectively, before bleaching and B(t) and Cell(t) intensities of the same regions at each time point after bleaching. The resulting F-values were further normalized to zero. EasyFRAP^33^ FRAP analysis software was used to fit single exponential curves to each series individually to calculate mobile fraction and half-recovery times for each region.

### Traction force microscopy

Traction force was measured as described previously by Qi *et al*.^18^. Briefly, glass-bottom dishes were silanized by 0.5% (3-Aminopropyl) triethoxysilane and activated by 0.5% glutaraldehyde. A drop of gel solution containing acrylamide (6%), bis-acrylamide (0.75%), ammonium persulfate, TEMED, and FluoSpheres® carboxylate-modified beads (diameter 0.2 μm, 1:85 dilution by volume) was added to the dishes and covered by a coverslip. The coverslip was removed and the gels were activated with Sulfo-SANPAH under UVA exposure, followed by conjugation with fibronectin (200 µg/ml). Talin1-null US OS cells were transiently transfected with wild type talin-1 or destabilized talin mutants and plated on the gels for 12 h. Traction force was measured as described previously^34^, using a Nikon A1 confocal microscope in Lexington VA Medical Center, Kentucky.

### Migration analysis

Polystyrene well plates were coated with 10 µg/ml fibronectin at 37 °C for 1 h and washed two times with PBS (pH 7.4). Transfected *TLN1*−/−*TLN2*−/− MEF cells were allowed to recover for 24 h, trypsinized and plated onto fibronectin coated well plates at a low confluency. Cells were allowed to attach for 1 h, followed by washing the cells with warm PBS (pH 7.4) to remove unbound (non-transfected) cells. The cells were cultured in fresh media for 30 min before imaging was started. Evos FL Auto (Life Technologies, USA) equipped with 37 °C and 5% CO_2_ incubator was used for live cell imaging for 12 hours at 120 second intervals. The resulting image stacks were analyzed with ImageJ version 1.50e with MTrackJ plugin^35, 36^.

### Microcontact printing of patterned substrates

Stamps were incubated for 10 minutes with a fibronectin (Fn) solution consisting of 45 µg/ml inactive human Fn (thermal denaturation at 90°), 5 µg/ml active human Fn (Sigma Aldrich) and 1 µg/ml Alexa Fluor 647-labeled bovine Fn (protein-labeling kit; Life Technologies). Fn molecules adsorbed to the stamp were transferred to a glass coverslip for 10 minutes. Stamped structures were incubated with 5 µg/ml human Vn (Sigma Aldrich) for 1 h to coat protein-free regions. Micropatterned substrates were rinsed once with 1 × PBS before they were used for cell culture. Transiently transfected wild type MEF cells were seeded and cultured for 2 h on Fn/Vn substrates in serum-free DMEM. After 2 h of cultivation cells were fixed and immunostained using anti-paxillin primary antibody (BD Bioscience) and Cy3-conjugated secondary antibody (Dianova).

### Structured illumination microscopy (SIM) and talin-1 localization analysis

SIM was performed at room temperature on a nonserial prototype microscope (ELYRA PS. 1; Carl Zeiss Microscopy) in superresolution SIM mode using a Plan Apochromat 63 × 1.40 NA oil objective (Carl Zeiss Microscopy). For each image, 15 raw images were processed and reconstructed to extract the superresolution information. Channels were aligned in x,y using predetermined shifts to correct for thermal drift or concussion.

SIM images of talin-1-GFP, paxillin label (Cy3) and labeled Fn (Alexa Fluor 647) were analyzed using Mander’s overlap coefficient to determine colocalization of talin-1 with Fn patches. A paxillin mask was created to remove cytosolic fluorescence signal caused by talin expression and to minimize its bias on the quantification (see Supplementary Figure S6). Paxillin masking was done by extending the paxillin SIM signal using Gaussian Blur function in ImageJ and by thresholding the resulting image. This mask was used to calculate Mander’s overlap coefficient with manual threshold to determine the degree of GFP fluorescence at sites of Alexa Fluor 647-labeled Fn. Data were obtained from three independent experiments (n = 30–40 cells per condition). For analyzing the colocalization of talin mutants and β3 and β1 integrin subtypes, masks based on widefield fluorescence images was used (see Supplementary Figure S6).

## Electronic supplementary material

Supplementary Information

Supplementary Movie S5
