# Supplementary material for: Mechanical stability of talin rod controls cell migration and substrate sensing
Source: Sci Rep. 2017 Jun 15;7:3571. doi: 10.1038/s41598-017-03335-2 (PMC5472591; doi:10.1038/s41598-017-03335-2)
Supplement: Supplementary file 1 — Supplementary Information [file 41598_2017_3335_MOESM1_ESM.pdf]

Mechanical stability of talin rod controls cell migration and substrate sensing

Rolle Rahikainen [1], Magdaléna von Essen [†1], Markus Schaefer [2], Lei Qi [3], Latifeh Azizi [1], Conor Kelly [1], Teemu O. Ihalainen [1], Bernhard Wehrle-Haller [4], Martin Bastmeyer [2], Cai Huang [3], Vesa P. Hytönen [1*].

1) Faculty of Medicine and Life Sciences and BioMediTech, University of Tampere, Finland and Fimlab Laboratories, Tampere, Finland

2) Zoological Institute, Cell- and Neurobiology, Karlsruhe Institute of Technology (KIT), Institute of Functional Interfaces (IFG), Karlsruhe, Germany

3) Markey Cancer Center and Department of Molecular and Biomedical Pharmacology, University of Kentucky, Lexington, KY, USA

4) Department of Cell Physiology and Metabolism, University of Geneva, Switzerland

† Co-first author

* Corresponding author, email: vesa.hytonen@uta.fi

Supplementary information

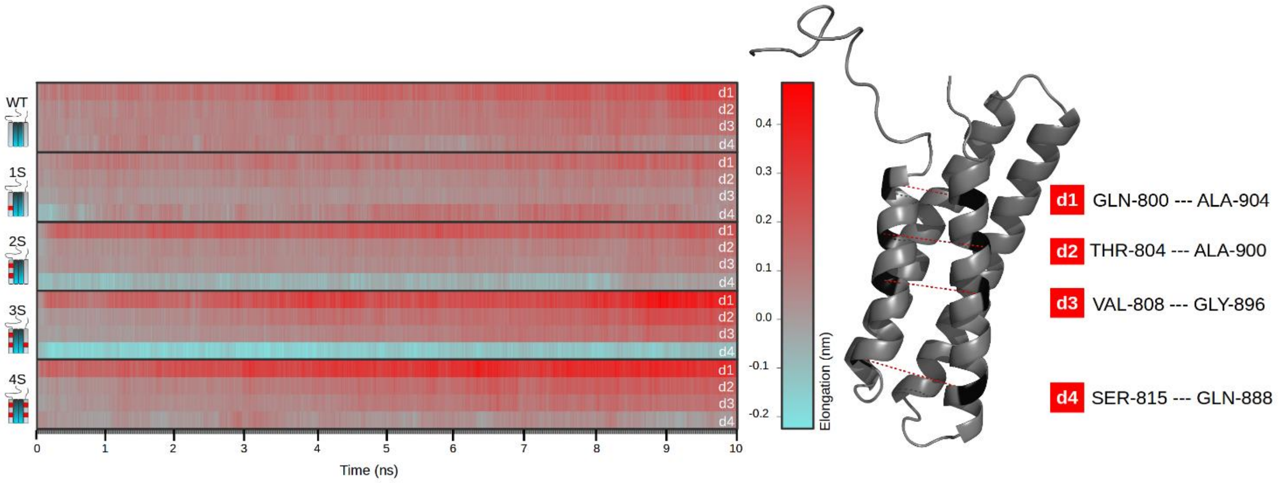

Supplementary figure S1. Talin R3 subdomain helix 1 (H1) to helix 4 (H4) distance during simulated stretching of the subdomain at 150 pN. H1 to H4 distance was analyzed at four vectors (d1 - d4), illustrated as dashed red lines on the structural model. In the graphical presentation of the d1 - d4 distances, decreased and increased distances are indicated with cyan and red colors, respectively.

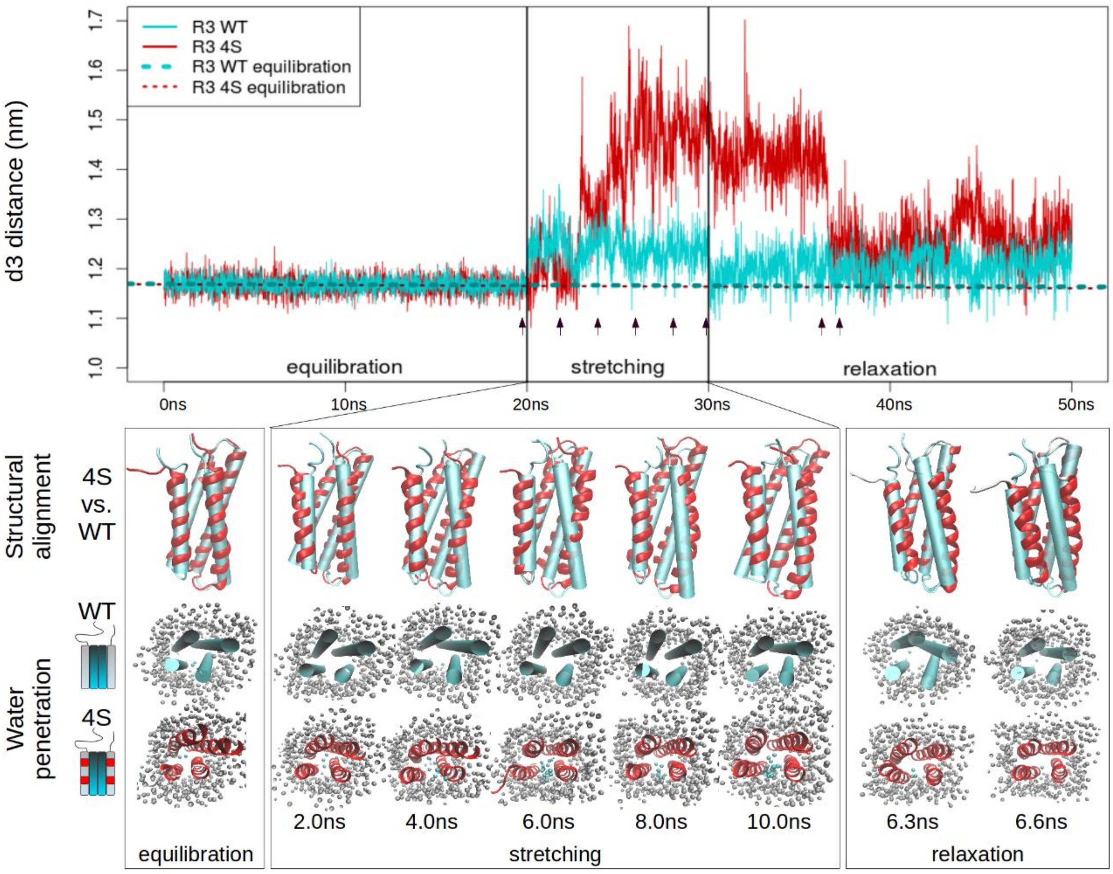

Supplementary figure S2. Domain refolding for wild type talin-1 R3 subdomain and destabilized 4S form. Starting from the structure coordinates after 10 ns pulling, simulations for domain refolding were performed at equilibration conditions for 20 ns. Top: The d3 (Val808-Gly896) distances for wild-type R3 subdomain (cyan solid line) and destabilized 4S form (red solid line) during equilibration, stretching and relaxation were plotted as a function of time. Cyan and red dashed lines indicate the average d3 distances during the equilibration step. Middle: Structural alignments for the wild-type R3 subdomain (cyan cylinder) and 4S mutant (red helices) during each step. Bottom: Water penetration into the R3 subdomain helix bundle core during stretching and relaxation. Note the exclusion of water from the core of the 4S form during the relaxation step.

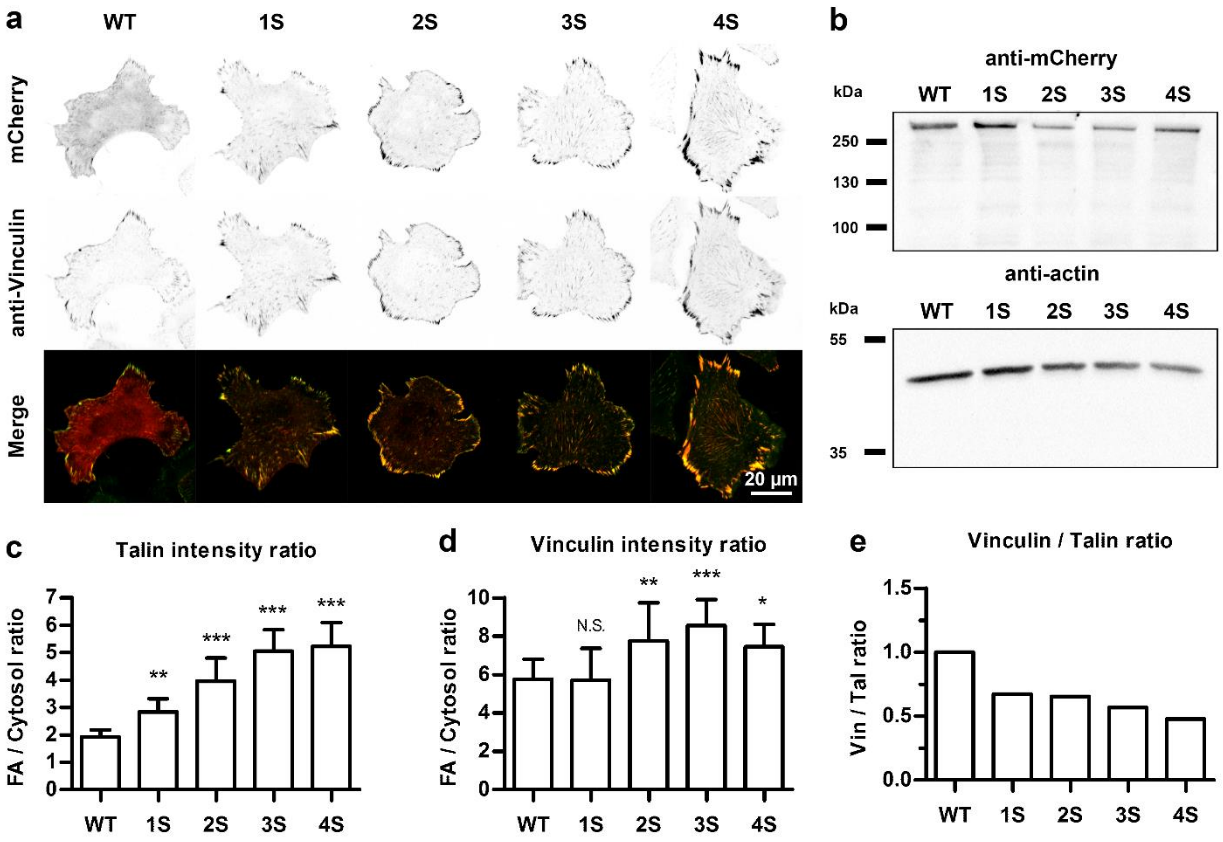

Supplementary figure S3. Talin-1 accumulation into focal adhesions in TLN1-/-TLN2-/- MEF cells. **(a)** Representative images of TLN1-/-TLN2-/- cells expressing mCherry-tagged talin-1 proteins. In merged images, mCherry signal is shown in red and vinculin antibody staining in green. Co-localization of the two signals is indicated by yellow color. Scale bar is 20 µm. **(b)** Western blot for concentration-normalized lysates of TLN1-/-TLN2-/- MEF cells expressing mCherry-tagged talin-1 proteins. Expressed talin proteins were detected by anti-mCherry antibody, while anti-actin antibody was used as a loading control. After normalization to actin intensity, the population level expression levels of destabilized 1S, 2S, 3S and 4S forms were 113%, 65%, 75% and 137% of the expression level of wild type talin. **(c)** Mean ratios of mCherry signal intensities measured from adhesion sites and cytosolic areas in cells expressing mCherry-tagged talin-1 mutant proteins. Error bars represent SD. n = 16 cells for each talin construct. **(d)** Mean adhesion/cytosolic signal ratios for vinculin antibody staining of cells expressing mCherry-tagged talin-1 forms. Error bars represent SD. n = 16 cells for each talin construct. In (c) and (d), The intensity ratios measured for the talin-1 mutants were compared to mCherry-tagged wild type talin-1 by One-way ANOVA and Tukey's test. *** = $p < 0.0001$ ** = $p < 0.001$, * = $p < 0.05$. **(e)** Vinculin/talin ratios for the results in (c) and (d).

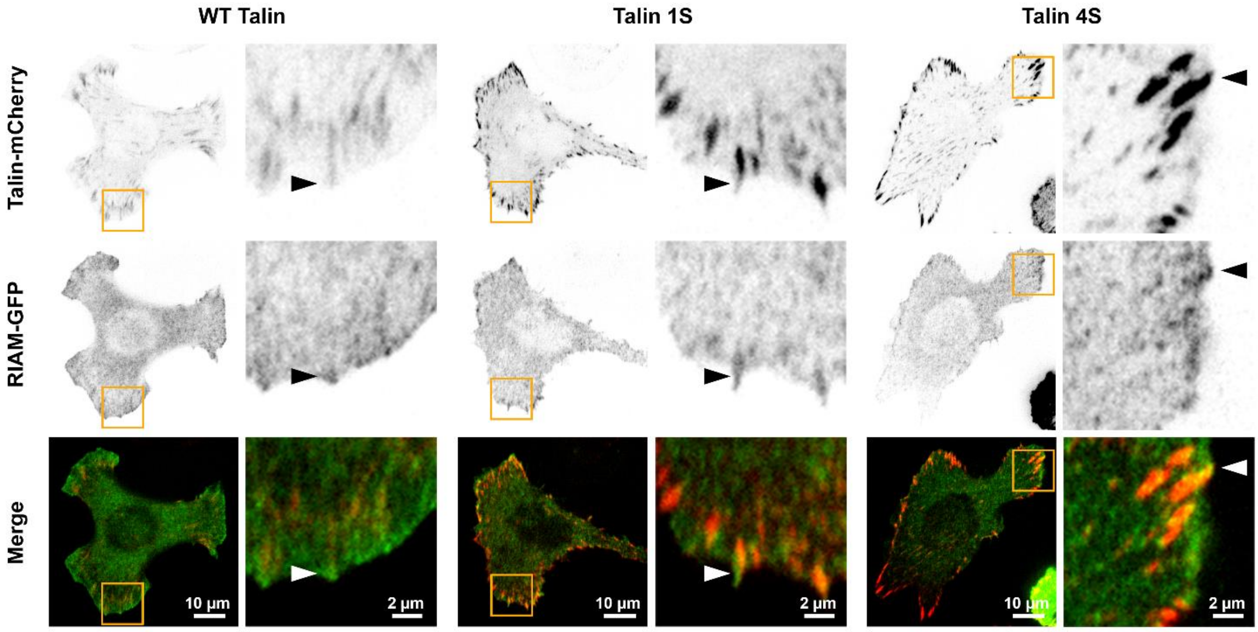

Supplementary figure S4. Localization of RIAM-GFP in cell expressing destabilized talin-1 proteins. TLN1-/-TLN2-/- cells were co-transfected with RIAM-GFP and mCherry-tagged wild-type talin-1 or 1S or 4S destabilized talin constructs. Co-transfected cells were plated on fibronectin coated glass for 120 min before fixation. Representative confocal microscope images (n = 10 for each talin construct) of the fixed cells are shown in the image panel. In the magnified views of lamellipodia, arrowheads indicate areas of nascent adhesion structures with high RIAM-GFP fluorescence signal and low talin-mCherry fluorescence signal. Co-expression of destabilized 1S or 4S mutants did not noticeably affect the localization of RIAM-GFP to the leading edge of advancing lamellipodia.

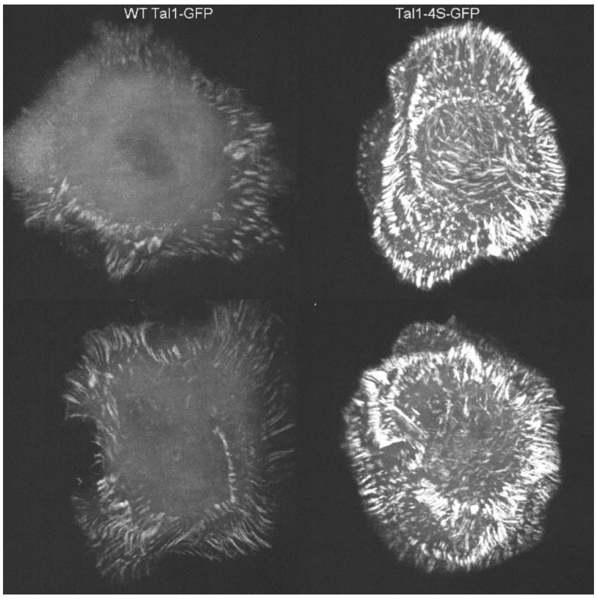

Supplementary movie S5. Live cell microscopy time-lapse video for TNL1-/-TLN2-/- cells expressing wild-type talin-1 or mechanically destabilized talin-1 4S form. Cells transfected with each construct were plated on fibronectin coated coverslips, allowed to attach for 15 min and imaged at 60 s intervals for 120 min. All imaging parameters were kept constant for all images to allow comparison of adhesion intensities.

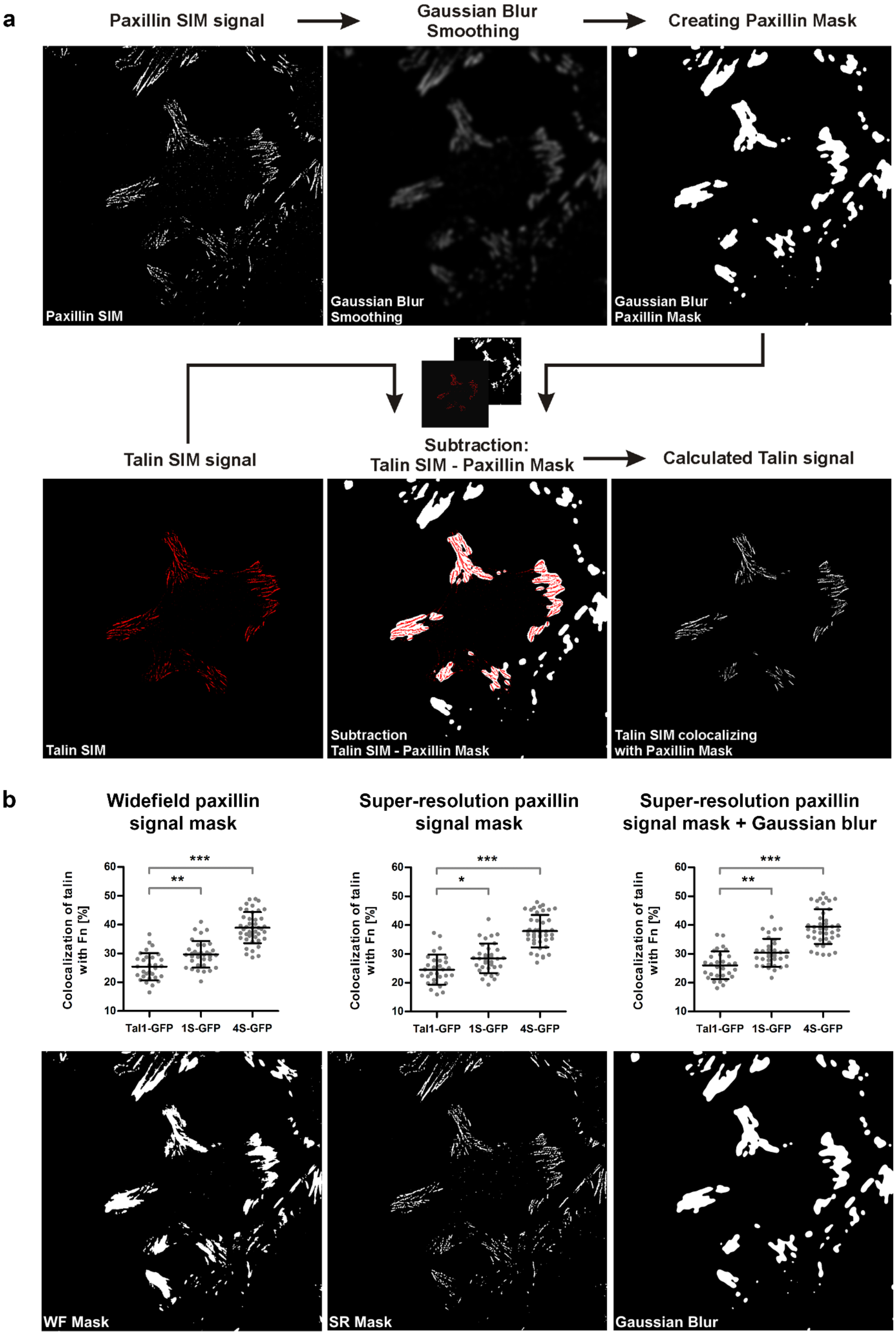
a
Paxillin SIM signal
Gaussian Blur Smoothing
Creating Paxillin Mask
Paxillin SIM
Gaussian Blur Smoothing
Gaussian Blur Paxillin Mask
Talin SIM signal
Subtraction: Talin SIM - Paxillin Mask
Calculated Talin signal
Talin SIM
Subtraction Talin SIM - Paxillin Mask
Talin SIM colocalizing with Paxillin Mask
b
Widefield paxillin signal mask
Super-resolution paxillin signal mask
Super-resolution paxillin signal mask + Gaussian blur
Colocalization of talin with Fn [%]
60
50
40
30
20
10
***
**
*
Tal1-GFP
1S-GFP
4S-GFP
WF Mask
SR Mask
Gaussian Blur

Supplementary figure S6. Paxillin masking and localization analysis for cells expressing talin-1 constructs on Fn/Vn patterned substrates. Transiently transfected wild type MEF cells were cultured for 2h on micropatterned Fn/Vn substrates, PFA-fixed and immunostained for paxillin. Analysis of superresolution microscopy (SIM) images was performed using imageJ to determine the degree of GFP fluorescence at sites of Alexa Fluor 647-labeled Fn with Mander's overlap coefficient. **a)** A Paxillin mask was created in order to remove unspecific cytosolic fluorescence signals from the cytosolic fraction of talin-GFP and thus focusing the analysis on high density talin clusters. For paxillin masking, the paxillin SIM signal was extended via Gaussian blur smoothing to focus on true talin-1-positive adhesions without biasing analysis towards absolute talin-1 localization with paxillin. Finally, analysis of talin-1 localization with fibronectin coating was performed with Mander's overlap coefficient using manual threshold. **b)** Comparison of results from talin localization analysis using three different types of masks based on the paxillin signal. Talin-1 signal masking using paxillin widefield signal (left hand side), paxillin super-resolution signal (Middle) or Gaussian blur smoothened paxillin super-resolution signal (right hand side) all yielded closely matching results, suggesting that masking the talin-1 signal before the analysis caused only negligible bias to the results. Datapoints were pooled from three fully independent experiments, n = 30, 34 and 43 for cells expressing wild type talin-1-GFP, 1S-GFP or 4S-GFP mutant, respectively. Statistical analysis by one-way ANOVA and Tukey's test. * = $p < 0.05$, ** = $p < 0.001$, *** = $p < 0.0001$.
